# Genomic Insights Into Sclerotinia Basal Stalk Rot Resistance Introgressed From Wild *Helianthus praecox* Into Cultivated Sunflower (*Helianthus annuus* L.)

**DOI:** 10.3389/fpls.2022.840954

**Published:** 2022-05-18

**Authors:** Zahirul I. Talukder, William Underwood, Christopher G. Misar, Gerald J. Seiler, Xiwen Cai, Xuehui Li, Lili Qi

**Affiliations:** ^1^Department of Plant Sciences, North Dakota State University, Fargo, ND, United States; ^2^USDA-Agricultural Research Service, Edward T. Schafer Agricultural Research Center, Fargo, ND, United States

**Keywords:** sunflower, *Helianthus praecox*, *Sclerotinia sclerotiorum*, basal stalk rot resistance, quantitative trait loci, QTL mapping

## Abstract

Crop wild relatives of the cultivated sunflower (*Helianthus annuus* L.) are a valuable resource for its sustainable production. *Helianthus praecox* ssp. *runyonii* is a wild sunflower known for its resistance against diseases caused by the fungus, *Sclerotinia sclerotiorum* (Lib.) de Bary, which infects over 400 broadleaf hosts including many important food crops. The objective of this research was to dissect the Sclerotinia basal stalk rot (BSR) resistance introgressed from *H. praecox* ssp. *runyonii* into cultivated sunflower. An advanced backcross quantitative trait loci (AB-QTL) mapping population was developed from the cross of a *H. praecox* accession with cultivated sunflower lines. The AB-QTL population was evaluated for BSR resistance in the field during the summers of 2017–2018 and in the greenhouse in the spring of 2018. Highly significant genetic variations (*p* < 0.001) were observed for the BSR disease in the field and greenhouse with a moderately high broad-sense heritability (*H*^2^) ranging from 0.66 to 0.73. Genotyping-by-sequencing approach was used to genotype the parents and the progeny lines of the AB-QTL population. A genetic linkage map spanning 1,802.95 cM was constructed using 1,755 single nucleotide polymorphism (SNP) markers mapped on 17 sunflower chromosomes. A total of 19 BSR resistance QTL were detected on nine sunflower chromosomes, each explaining 2.21%–16.99% of the phenotypic variance for resistance in the AB-QTL population. Sixteen of the 19 QTL had alleles conferring increased BSR resistance derived from the *H. praecox* parent. SNP markers flanking the identified QTL will facilitate marker-assisted breeding to combat the disease in sunflower.

## Introduction

Basal stalk rot (BSR) is an important disease of sunflower (*Helianthus annuus* L.) in the cool and humid growing environment causing serious economic damage to the crop worldwide (Gulya et al., 1997). The disease is caused by the necrotrophic fungal pathogen, *Sclerotinia sclerotiorum* (Lib) de Bary, which infects over 400 broadleaf hosts including many important food crops (Boland and Hall, 1994; Bolton et al., 2006). In the United States alone, hundreds of millions of dollars’ worth of economic losses occur every year due to diseases caused by the *S. sclerotiorum* (Bolton et al., 2006). Throughout the entire growing season, sunflower suffers from three major forms of the disease caused by the fungus, BSR, also called Sclerotinia wilt, mid stalk rot (MSR), and head rot (HR). Under favorable growth conditions, the sclerotia (the compact mass of hardened fungal mycelium) germinates beneath the soil and the growing mycelia invade the sunflower roots to incite the BSR disease (unique to sunflower), in contrast, both MSR and HR are caused by the germination of airborne ascospore released from the apothecia and carried by wind and rain splashing on the sunflower leaves and capitula (Gulya et al., 1997).

The Northern Great Plains states of North Dakota, South Dakota, and Minnesota are the major sunflower production states of the United States, which together produced over 87% of the total sunflower in 2021 (USDA, 2021). Sclerotinia BSR is one of the predominant diseases of sunflower in this region (Gulya et al., 1989; Seiler et al., 2017a). The climatic condition of this region is highly favorable for BSR disease development during the sunflower growing season and an outbreak of the disease can cause serious economic loss for the growers (Gulya et al., 1989). Chemical and biological control of the BSR disease is limited and often not feasible due to the soilborne nature of the disease. Use of host genetic resistance is the most economical and effective approach for BSR management and has been used effectively in many breeding programs. Sunflower genetic resources are routinely evaluated for novel sources of BSR resistance in the cultivated and wild gene pools (Block et al., 2009, 2010, 2012; Talukder et al., 2014b; Seiler et al., 2017a).

Sclerotinia BSR resistance in sunflower is a polygenic trait governed by multiple small effect genes (Talukder et al., 2014c, 2016, 2021). Quantitative genetic variations for BSR resistance have been mapped in different segregating populations developed from both cultivated and wild sunflower species (Davar et al., 2010; Amouzadeh et al., 2013; Talukder et al., 2014c, 2016, 2021; Paknia et al., 2020). In the growth chamber under a controlled environment, Davar et al. (2010) identified seven QTL associated with BSR resistance on linkage groups (LGs) 1, 2, 4, 6, 8, 14, and 17 in a recombinant inbred line (RIL) population developed from the cross of PAC1/RHA 266, while Amouzadeh et al. (2013) identified five QTL on LGs 1, 3, 8, 10, and 17 in the same population using a different fungal isolate. Later, the population was evaluated in the field identifying five genomic regions associated with BSR resistance, two each on LGs 11 and 15, and one on LG14 (Paknia et al., 2020). Proportions of the phenotypic variance explained by each of the QTL in the controlled growth chamber study were in the range of 0.5%–8%, while they were higher for some QTL in the field study, ranging from 1.9% to 37.3% of the total variation. Six BSR resistance QTL were identified on LGs 4, 9, 10, 11, 16, and 17 in a RIL population derived from the cross HA 441/RHA 439 evaluated in the field for multiple years and locations (Talukder et al., 2016). The QTL on LGs 10 and 17 were the most stable across years and locations and explained 31.6% and 20.2%, respectively of the total phenotypic variations. Using a worldwide collection of open pollinated sunflower lines, Talukder et al. (2014c) identified two genes, *HaCOI1*-1 and *HaCOI1*-2, on LG14 strongly associated with Sclerotinia BSR resistance and the two genes are orthologous and paralogous to the *Arabidopsis thaliana COI*1 gene, respectively.

Sunflower is one of the few crop species that originated and was domesticated in eastern North America (Blackman et al., 2011). There are 53 species for the genus *Helianthus* known to date, including 14 annual and 39 perennial species (Seiler et al., 2017b). All annual *Helianthus* species are diploid (2n = 2x = 34) while the perennial species have varied ploidy levels ranging from diploid to hexaploid with a few mixaploid species (Kane et al., 2013; Seiler et al., 2017b). Wild *Helianthus* species serve as potential sources of novel genes for several desirable traits, such as resistance to biotic and abiotic stresses, cytoplasmic male sterility, fertility restorer genes and other agronomic traits. Several of the novel genes from wild relatives have been utilized in cultivated sunflower development (for review, see Seiler et al., 2017b). Despite reports of high levels of Sclerotinia resistance existing in the secondary gene-pool, comprehensive studies have not been pursued to characterize and exploit the resistance in practical sunflower breeding programs. Only recently, introgression and molecular mapping of Sclerotinia resistance from wild species have been reported and germplasms with introgressed resistance have been released (Qi et al., 2016, 2018; Talukder et al., 2019a,b, 2021). Talukder et al. (2021) mapped 21 BSR resistance QTL in an advanced backcross (AB-QTL) population developed from the cross of HA 89 and an accession of the wild annual species *H. argophyllus* Torr. & Gray (silverleaf sunflower) using a high-density single nucleotide polymorphism (SNP) linkage map. The detected QTL were distributed on 11 sunflower chromosomes (LGs 2, 5, 6, 7, 8, 9, 10, 11, 14, 16, and 17) and each QTL explaining between 4.5% and 22.6% of the phenotypic variance. Remarkably, 13 of the 21 QTL had BSR resistance alleles derived from the *H. argophyllus* parent (Talukder et al., 2021).

*Helianthus praecox* Engelm. & A. Gray is a wild annual sunflower species with three subspecies: ssp. *praecox*, ssp. *runyonii*, and ssp. *hirtus* widespread in the state of Texas (Schilling, 2006; Turner, 2014). *Helianthus praecox* has previously been shown to possess high levels of resistance against Sclerotinia diseases (Škorić, 1987; Seiler, 1994, 2010; Rönicke et al., 2004; Christov and Velasco, 2008; Block et al., 2009, 2010; Christov, 2013). An AB-QTL population (Tanksley and Nelson, 1996) was developed by crossing and backcrossing of a *H. praecox* ssp. *runyonii* accession with cultivated sunflower lines. The objective for this study was to evaluate the population in the field and greenhouse and perform an in-depth inheritance analysis of BSR resistance segregating in the population using a high-density genetic map.

## Materials and Methods

### Plant Materials

A wild annual *H. praecox* subsp. *runyonii* accession (PI 468853) was used as the BSR resistance donor parent for this study. The wild species accession was collected in Texas, United States, previously characterized as highly resistant against Sclerotinia BSR disease in greenhouse tests (Block et al., 2009, 2010) and used to develop the BSR resistant introgression lines HA-BSR6, HA-BSR7 and HA-BSR8 (Talukder et al., 2019a). PI 468853 was crossed and backcrossed with two cultivated sunflower inbred lines, HA 89 and HA 458, to develop an AB-QTL mapping population comprised of 174 BC_2_F_4_ families. The initial cross was made with the nuclear male sterile (NMS) version of HA 89 (PI 559477) followed by a cross with sunflower inbreed line HA 458 (PI 655009) and a backcross with the male-fertile version of HA 89 (PI 599773). HA 89 is an oilseed maintainer line with good agronomics but lacks resistance to BSR. The NMS HA 89 is a mutant developed by streptomycin treatment of HA 89, which possess a recessive gene, *ms9* that controls male sterility (Jan and Rutger, 1988). HA 458 is a high oleic inbred line characterized by good agronomic traits and downy mildew resistance released in 2010 (Hulke et al., 2010; Qi et al., 2015).

### Experimental Design and Phenotypic Evaluation

#### Field Evaluation

A total of 174 BC_2_F_4_ families, the cultivated sunflower parental line HA 89, and two sunflower hybrids Cargill 272 and Croplan 305 representing the susceptible and resistant checks, respectively were evaluated for Sclerotinia BSR resistance at the Carrington Research Extension Center, North Dakota State University, ND (47.4495^O^ N, 99.1263^O^ W) during the summers of 2017–2018. The trials were conducted using a randomized complete block design with three replications. Each entry line was planted with 30 seeds in 6-m long single-row plot and row spacing was maintained at 75 cm. The trials were inoculated at R1 growth stage (Schneiter and Miller, 1981) with *S. sclerotiorum*-infested millet inoculum prepared from isolate NEB-274. This isolate was collected in the Great Plains region of the United States and is aggressive in causing disease on sunflower. Approximately, 90 g per row of the inoculum was placed in furrows at 5–7 cm depth and 15–20 cm away from each row using a granular chemical applicator drawn by a tractor (Gulya et al., 2008). The typical BSR symptom develops near the base of the sunflower stalk at the soil line with characteristic tan to manila colored, water-soaked lesion girdling the stalk with occasionally visible white mycelium. Disease incidence (DI) was scored at approximately 10 weeks after inoculation as the percentage of plants exhibiting typical BSR symptoms.

#### Greenhouse Evaluation

The mapping population was evaluated for BSR resistance in the greenhouse in 2018 with three replications. All 174 BC_2_F_4_ families along with the recurrent parent, HA 89 and a resistant inbred line, RHA 801 were grown in 32-well 3-inch-deep sheet pots (#730654C, T.O. plastics, Clearwater, MN). Each sheet pot contains eight rows of four wells with a total dimension of 21.06 inch × 10.56 inch. The wells of the sheet pots were filled with potting mix (Metro Mix 902, Sun Gro Horticulture, Agawam, MA) and placed in compatible open flats (#710245C, T.O. plastics, Clearwater, MN). Sunflower seeds were planted in sheet pots (single seed per well) and the flats were randomly arranged on greenhouse benches. The temperature of the greenhouse was maintained at ~22°C and supplemental lighting was provided to maintain a 16-h photoperiod. Three experimental replications were conducted in which a total of 12 plants per entry were inoculated for BSR evaluation in each replication. Inoculation was performed by removing rootbound sunflower plants from pots, transferring 0.76 g *S. sclerotiorum* infested millet inoculum into the bottom of the pot, and returning the plant to the pot to place the root-mass in direct contact with mycelial inoculum. Inoculated plants were then arranged on greenhouse benches following completely randomized design in 12 blocks and were provided sufficient moisture for disease development.

### Inoculum Preparation

The method of *S. sclerotiorum* inoculum preparation for BSR evaluation of both field and greenhouse trials has been described elsewhere (Underwood et al., 2021). In brief, single sterile sclerotia of *Sclerotinia sclerotiorum* isolate NEB-274 was grown on potato dextrose agar (PDA) plates. Plugs from the growing edge of the mycelial colony were then cut using a cork borer, transferred to five new PDA plates, and allowed to grow for another 4 days at 21°C. The *S. sclerotiorum* cultures were then cut into pieces and stirred into autoclaved white proso millet under sterile conditions. The infested millet seed was stored at 21°C for 6 days to allow colonization by the *S. sclerotiorum* mycelium. The *S. sclerotiorum* inoculated millet seed was then dried at 35°C for 5 days and stored in plastic containers at 4°C until needed.

### Data Collection and Statistical Analysis

In the Carrington field trials, DI data were collected at the physiological maturity stage (R9) of the plants as the percentage of plants showing typical BSR symptoms with basal stem lesions. An analysis of variance (ANOVA) of the DI data was performed across both years using PROC MIXED in SAS version 9.4 (SAS Institute, 2016). All factors were treated as random effects, except genotype, using the model: $y_{ijk}=\mu +l_{i}+b{\left(l\right)}_{ij}+g_{k}+gl_{ik}+e_{ijk}$ , where y is the DI of the *k^th^* genotype tested in the *j^th^* replication of the *i^th^* year, μ is the overall mean, $l_{i}$ is the effect of the *i^th^* year, $b{\left(l\right)}_{ij}$ is the effect of the *j^th^* replication nested in the *i^th^* year, $g_{k}$ is the genetic effect of the *k^th^* genotype, $gl_{ik}$ is the interaction effect of the *k^th^* genotype and *i^th^* year, and $e_{ijk}$ is the random experimental error. Broad-sense heritability on an entry mean basis was estimated following Nyquist (1991) as: $H^{2}=\sigma_{g}^{2}/\left(\sigma_{g}^{2}+\sigma_{ge}^{2}/l+\sigma_{e}^{2}/lr\right)$ , where $\sigma_{g}^{2}$ is the genetic variance, $\sigma_{ge}^{2}$ is the genotype x year variance, $\sigma_{e}^{2}$ is the error variance, *r* is the number of replications, and *l* is the number of years.

In the greenhouse, individual plants were evaluated daily for 28 days post-inoculation (dpi) for terminal wilting or whole plant desiccation indicative of death due to BSR (Underwood et al., 2021) and days to plant death was recoded to calculate area under the disease progress curve (AUDPC) and BSR disease rating (DR). AUDPC was calculated following Shaner and Finney (1977) as:


$$\mathrm{AUDPC}=\overset{n}{\underset{i=1}{\sum }}\left[\frac{\left(y_{i}+y_{i+1}\right)}{2}\right]\left(t_{i+1}-t_{i}\right)$$

where $y_{i}$ is the proportion of dead plants at the *i*^th^ observation, *t* is the time (dpi) at the *i*^th^ observation, and n is the total number of observations on which BSR was recorded. ANOVA of the AUDPC and DR data were performed using PROC MIXED in SAS version 9.4 (SAS Institute, 2016), where genotype was considered as fixed effect. Broad-sense heritability on an entry mean basis was estimated following Nyquist (1991) as: $H^{2}=\sigma_{g}^{2}/\left(\sigma_{g}^{2}+\sigma_{e}^{2}/r\right)$ , where $\sigma_{g}^{2}$ is the genetic variance, $\sigma_{e}^{2}$ is the error variance, *r* is the number of replications. Spearman’s rank correlations (*ρ*) were performed for BSR disease data among lines of the HA 89/*H. praecox* AB-QTL population tested in both field and greenhouse during 2017–2018 using the statistical package R version 4.1.1 (R Core Team, 2021).

### DNA Extraction and Single Nucleotide Polymorphism Genotyping

Leaf tissues of the parents and the 174 BC_2_F_4_ families of the HA 89/*H. praecox* AB-QTL population were collected and freeze-dried from 3 to 4 weeks old sunflower seedlings. DNA of each BC_2_F_4_ family was buked from four individual plants. Approximately, 50 mg of freeze-dried leaf tissue per line were used to extract genomic DNA using Qiagen DNeasy 96 plant kit (Qiagen, Valencia, CA, United States) with a minor modification of the manufacturer’s protocol following Horne et al. (2004). The quality and the quantity of the extracted DNA were determined with a NanoDrop 2000 Spectrophotometer (Thermo Fisher Scientific). Genotyping of the population was conducted by LGC Genomics GmbH (Berlin, Germany) using genotyping-by-sequencing (GBS) technology. Libraries were prepared with *MsII* enzyme digestion and an Illumina NextSeq 500 V2 platform was then used for sequencing (150 bp paired end). GBS reads were mapped, and SNP calling was performed against the sunflower reference genome HanXRQr1.0 (Badouin et al., 2017).^1^ A total 3,150 good-quality variants (SNPs) were obtained for linkage mapping. The SNP markers were given a prefix of “C” with their respective chromosome numbers (1–17) that correspond to the 17 sunflower chromosomes followed by a number being the physical position of the SNP on the HanXRQr1.0 genome assembly. A 400 bp sequence surrounding each of the SNP significantly associated with the BSR-resistance QTL in the current study is presented in Supplementary Table 1.

### Linkage Mapping

Linkage analysis was performed using the JoinMap 4.1 software (Stam, 1993; Van Ooijen, 2006). The markers were first tested for goodness-of-fit to the expected segregation ratio using the chi-square test option of the software. Markers with extreme segregation distortion were removed for linkage analysis. Markers with identical genotypes (similarity value = 1.000) were excluded from linkage analysis momentarily using “similarity of loci” option of the software. These co-segregating markers will map at the same loci, however they put an additional load on the calculation effort and significantly slows down the analysis when handling a large dataset. Markers were assigned to LGs based on pairwise recombination frequencies with a logarithm of the odds (LOD) parameter values ranging from 3 to 10. The regression mapping algorithm option of the software was chosen to perform the linkage analysis. The “ripple” command was used each time after adding a marker to the LG and three mapping rounds were performed to finalize a linkage group. The recombination values were converted into a map distance (cM) using the Kosambi mapping function (Kosambi, 1943). Seventeen LGs corresponding to the 17 sunflower chromosomes were constructed using the SNP markers. The excluded “similarity of loci” markers was placed in the final map along with their corresponding co-segregating marker positions.

### QTL Mapping

The phenotypic data collected in both field and greenhouse trials were first assessed for normality using Shapiro–Wilk normality test (Shapiro and Wilk, 1965). Due to non-normal distributions, the best linear unbiased predictors (BLUP) were used for QTL analysis of both field and greenhouse BSR data. QTL analysis of BSR field DI was analyzed using data from individual years, as well as a combined analysis of 2 years data. The initial QTL analysis was performed using the composite interval mapping (CIM) option of WinQTL Cartographer v2.5 software (Zeng, 1994; Wang et al., 2012). In the CIM analysis, the scanning for BSR resistance QTL across the sunflower genome was performed using both the forward and backward regression method (model 6). The parameters were set for a window size of 10 cM with a walk speed of 1 cM and selecting up to five control markers for the analysis. Significance LOD threshold for each trait were determined independently using 1,000 times permutation tests (Churchill and Doerge, 1994). QTL analyses were also performed using QTL IciMapping v4.1 software (Meng et al., 2015) to compare the results obtained from WinQTL Cartographer. The inclusive composite interval mapping (ICIM) option of the software that reportedly proved more efficient for background control *via* a two-step mapping strategy (Li et al., 2007; Wang, 2009) was used for QTL analysis. In the first step, a stepwise regression is applied to identify most-significant regression variables, while in the second step, interval mapping is performed using phenotypes adjusted by the markers identified in the first step (Li et al., 2007; Wang, 2009). A 95% CI was used to estimate the QTL flanking region using 1-LOD of the most likely QTL peak position. Linkage maps along with the detected QTL were drawn using MapChart v2.2 (Voorrips, 2002). BSR resistance QTL identified in the current study were named following the convention proposed by Talukder et al. (2016). The naming of each QTL started with a prefix Q for QTL, a three-letter descriptor of the trait under study (BSR), the LG number, followed by a sequential number of the QTL so far identified in that LG for the trait.

## Results

### Field Evaluation of BSR Disease Incidence

**Figure 1:**
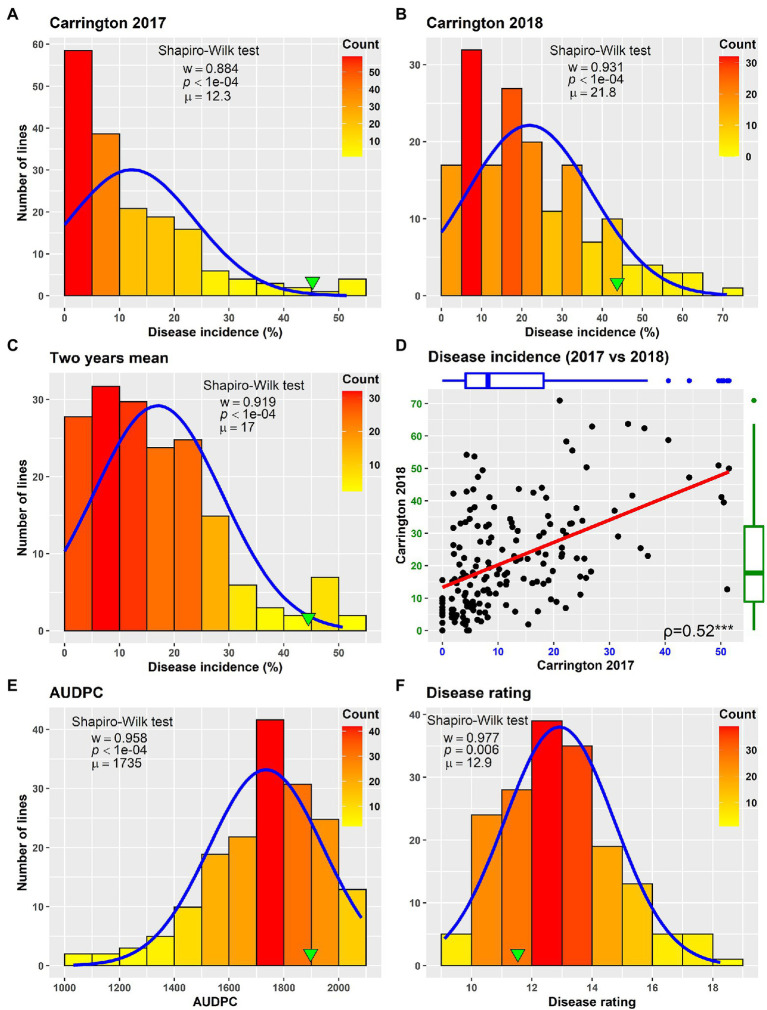
Frequency distribution of Sclerotinia basal stalk rot disease incidence **(A–D)**, area under disease progress curve **(E)** and BSR disease rating **(F)** in the HA 89/*Helianthus praecox* AB-QTL population evaluated in the field in Carrington, ND during 2017–2018 **(A–D)** and in the greenhouse in 2018 **(E,F)**. The green arrowheads indicate the value of the recurrent parent HA 89. The Shapiro–Wilk normality test statistic (*w*), the probability value (*p*), and the mean (*μ*) of the data for each environment are shown inside the respective plots.

Field evaluation of the HA 89/*H. praecox* AB-QTL population was performed at Carrington, ND during the summers of 2017–2018. The prevalence of BSR disease in the AB-QTL population was lower in 2017 than in 2018 with a mean disease incidence (DI) of 12.3% and 21.8%, respectively (Figure 1; Supplementary Figure 1). A wide range of BSR DI was observed for the HA 89/*H. praecox* progeny lines in both seasons, ranging from 0 to 51.5% and 0 to 71.0% DI, respectively, for the 2017 and 2018 Carrington trials. The mean DI across two seasons was 17.0%, ranging from 0% to 50.7%. Shapiro–Wilk test revealed that the DI data for both 2017 and 2018 seasons and the mean DI across the seasons were not normally distributed and skewed toward lower values (Figure 1). The BSR DI of the recurrent parent, HA 89 for the 2017 and 2018 seasons and for the mean across both seasons were 45.2%, 43.7%, and 44.5%, respectively. Analysis of variance (ANOVA) revealed highly significant (*p* < 0.001) genetic variations for BSR DI in both years (data not shown). Significant variations were also observed for the genotype and genotype × environment (G × E) interactions in the combined analysis (Table 1). However, no significant effect of the environment or the replications nested within environments was observed for the trait. Highly significant (*p* < 0.001) Spearman’s rank correlation (*ρ* = 0.52) was observed between BSR DI data for the 2017 and 2018 seasons (Figure 1; Table 2). The broad-sense heritability (*H*^2^) estimate of the BSR DI measured in the field across two seasons was 0.66.

**Table 1:** Combined analysis of variance for Sclerotinia basal stalk rot disease incidence scores among individuals of the HA 89/*Helianthus praecox* AB-QTL population tested at Carrington, ND during 2017–2018.

Component	*df*	Variance estimate	Confidence limit (0.05)		***F***/*Z* value^†^	*p* > ***F***/*Z*
			Lower	Upper		
Genotype (Gen)	173	$\sigma_{g}^{2}\;$ = 90.68	66.32	131.53	**2.90**	**<0.0001**
Environment (Env)	1	$\sigma_{l}^{2}\;$ = 43.48	8.23	96,733	0.67	0.2507
Rep (Env)	4	$\sigma_{r}^{2}\;$ = 5.74	1.76	101.68	1.15	0.1254
Gen × Env	172	$\sigma_{gl}^{2}\;$ = 21.84	9.93	81.03	2.23	0.0128
Error	689	$\sigma_{e}^{2}$ = 219.51	198.06	244.68		

*Coefficient of variations (CV) for DI in the field = 86.9%*.

† *In the PROC MIXED model, genotypes were considered fixed and, therefore, subject to F-test (values in bold). F, Fisher’s F-test statistic; Z, Z-test statistic*.

**Table 2:** Analysis of variance for area under disease progress curve (AUDPC) and Sclerotinia basal stalk rot disease rating (DR) scores measured among individuals of the HA 89/*Helianthus praecox* AB-QTL population tested in the greenhouse.

Trait	Component	*df*	Variance estimate	Confidence limit (0.05)		***F***/*Z* value^†^	*p* > ***F***/*Z*
				Lower	Upper		
AUDPC	Genotype	173	$\sigma_{g}^{2}\;$ = 31,230	23,728	42,973	**3.66**	**<0.0001**
	Rep	2	$\sigma_{r}^{2}\;$ = 103,208	27,929	4,126,994	1.00	0.1591
	Error	346	$\sigma_{e}^{2}$ = 35,216	30,505	41,114		
DR	Genotype	173	$\sigma_{g}^{2}\;$ = 2.328	1.7488	3.2541	**3.31**	**<0.0001**
	Rep	2	$\sigma_{r}^{2}\;$ = 3.650	0.9854	148.57	1.00	0.1598
	Error	346	$\sigma_{e}^{2}$ = 3.018	2.6146	3.5239		

*CV for the AUDPC = 10.8% and for DR = 13.5%. F, Fisher’s F-test statistic; Z, Z-test statistic*.

† *In the PROC MIXED model, genotypes were considered fixed and, therefore, subject to *F*-test (values in bold)*.

### Greenhouse Evaluation of BSR Resistance

Greenhouse evaluation of the area under disease progress curve (AUDPC) and BSR disease rating (DR) also showed continuous variation in the HA 89/*H. praecox* AB-QTL population, a typical characteristic of quantitative disease resistance (Figure 1; Supplementary Figure 1). The AUDPC data was skewed toward higher values with mean across three replications of 1,735, ranging from 1,032 to 2,083. The recurrent parent, HA 89 had an AUDPC value of 1,897. The distribution of DR data was also skewed, but toward lower values. The mean across three replications was 12.9 dpi (days post-inoculation), ranging from 9.1 to 18.3 dpi in the AB-QTL population. The recurrent parent, HA 89 had a DR value of 12.5 dpi. Statistical analysis revealed highly significant (*p* < 0.001) variations for both AUDPC and DR measured in the HA 89/*H. praecox* AB-QTL population in greenhouse trials (Table 3). The broad-sense heritability (*H*^2^) estimates for AUDPC and DR were 0.73 and 0.70, respectively, and the Spearman’s rank correlations (*ρ*) between these two traits was highly significant (*ρ* = 0.98, *p* < 0.001). Spearman’s rank correlations among the BSR resistances traits measured in the field and in the greenhouse were also significant at various levels (Table 2).

**Table 3:** Spearman’s rank correlations (*ρ*) between Sclerotinia basal stalk rot disease data among individuals of the HA 89/*Helianthus praecox* AB-QTL population tested in the field and in the greenhouse during 2017–2018.

Environment		Carrington 2017	Carrington 2018	AUDPC
Field	Carrington 2018	0.52^***^	–	–
Greenhouse	AUDPC	0.26^***^	0.19^**^	–
	Disease rating	0.22^**^	0.16^*^	0.98^***^

* *Significant at the 0.05 probability level*.

** *Significant at the 0.01 probability level*.

*** *Significant at the 0.001 probability level*.

### Genetic Linkage Mapping

Linkage analysis mapped a total of 1,755 SNP markers to 1,710 unique loci on 20 LGs. Markers on chromosomes 6, 7, and 11 produced two independent LGs, while markers of the 14 remaining chromosomes each organized into single LGs (Table 4). The detailed description of the HA 89/*H. praecox* AB-QTL population linkage map is presented in Supplementary Table 2. Only 2.6% (45 SNPs) of the total mapped SNP markers co-segregated with other SNPs in the linkage groups. The length and the number of mapped loci varied greatly across the LGs. Seven LGs had over 100 loci mapped with the highest on LG4 (192), followed by LG2 (183), LG16 (174), LG10 (157), and LG13 (134). The three chromosomes that split into two separate LGs each had the lowest number of mapped loci with 13, 15, 19, 30, 33 and 35, for LG7b, LG6b, LG7a, LG11b, LG6a and LG11a, respectively. The total length of the HA 89/*H. praecox* linkage map covered 1,802.95 cM with the longest LG13 (128.04 cM), followed by LG14 (122.50 cM), LG10 (117.56 cM), and LG2 (113.18 cM), and the shortest LG1 (73.71 cM) (Table 4). Overall, the density of the mapped loci across the sunflower genome was 1.05 cM/locus, ranging from 0.50 to 5.15 cM/locus on LG16 and LG6b, respectively. Over 96% of the gaps between two adjacent loci were less than 5 cM, while only 15 gaps throughout the genome were > 10 cM in the linkage map (Table 4).

**Table 4:** Summary of sunflower linkage map developed using SNP markers in an advanced backcross population derived from the cross of HA 89 and wild annual sunflower species, *Helianthus praecox*.

Linkage group	Map length (cM)	No. of loci	No. of markers	cM/Locus	cM/Marker	No. of large gaps	
						5–10 cM	>10 cM
LG1	73.712	82	82	0.90	0.90	3	0
LG2	113.182	183	189	0.62	0.60	1	1
LG3	109.082	67	70	1.63	1.56	3	0
LG4	101.918	192	195	0.53	0.52	5	0
LG5	86.916	62	62	1.40	1.40	2	0
LG6a	36.290	33	35	1.10	1.04	2	0
LG6b	77.242	15	16	5.15	4.83	4	3
LG7a	74.514	19	21	3.92	3.55	3	2
LG7b	63.295	13	13	4.87	4.87	4	2
LG8	89.704	93	95	0.96	0.94	3	1
LG9	108.662	120	122	0.91	0.89	0	0
LG10	117.558	157	162	0.75	0.73	1	1
LG11a	86.211	35	35	2.46	2.46	5	1
LG11b	40.466	30	30	1.35	1.35	0	0
LG12	90.038	76	77	1.18	1.17	2	1
LG13	128.041	134	139	0.96	0.92	3	1
LG14	122.500	105	109	1.17	1.12	5	0
LG15	84.620	54	58	1.57	1.46	2	0
LG16	87.157	174	177	0.50	0.49	2	1
LG17	111.845	66	68	1.69	1.64	4	1
Total	1802.953	1710	1755	1.05	1.03	54	15

### Quantitative Trait Loci Mapping

**Figure 2:**
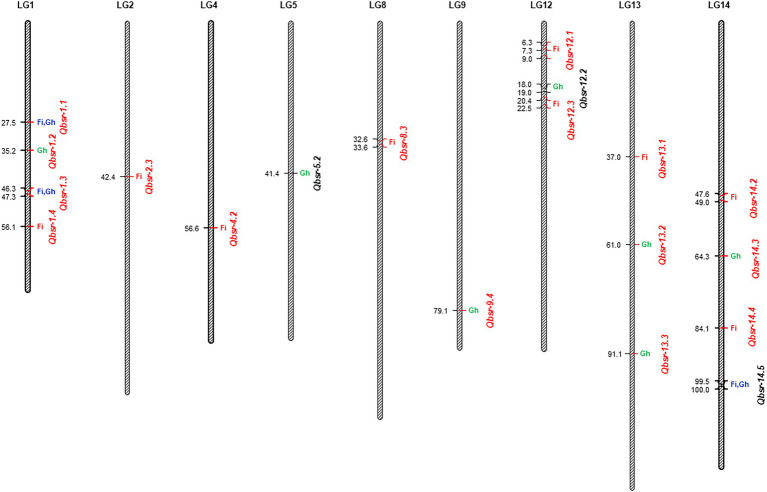
Quantitative trait loci (QTL) associated with Sclerotinia basal stalk rot (BSR) resistance identified in the HA 89/*Helianthus praecox* advanced backcross population. The QTL where BSR resistance alleles derived from the wild *Helianthus praecox* parent were indicated in red fonts. Fi, field environment; Gh, greenhouse environment.

A total of 19 BSR resistance QTL were identified on nine chromosomes in the HA 89/*H. praecox* AB-QTL population evaluated in the field and greenhouse environments (Table 5; Figure 2). The proportion of phenotypic variance explained by each detected QTL ranged from 2.21% to 16.99% in the AB-QTL population. Nine of these QTL, *Qbsr-1.4*, *Qbsr-2.3*, *Qbsr-4.2*, *Qbsr-8.3*, *Qbsr-12.1*, *Qbsr-12.3*, *Qbsr-13.1*, *Qbsr-14.2*, and *Qbsr-14.4*, were detected for BSR DI measured in the field; seven QTL, *Qbsr-1.2*, *Qbsr-5.2*, *Qbsr-9.4*, *Qbsr-12.2*, *Qbsr-13.2*, *Qbsr-13.3*, and *Qbsr-14.3*, were detected for the trait evaluated in the greenhouse, while only three QTL, *Qbsr-1.1*, *Qbsr-1.3*, and *Qbsr-14.5*, were detected from both field and greenhouse tests (Table 5). Sixteen of these QTL had BSR resistance alleles derived from the wild *H. praecox* parent, while the remaining three QTL had resistance alleles derived from the recurrent parents. The highest number of QTL were detected on LGs 1 and 14 with four QTL each. Three QTL were detected on each of the LGs 12 and 13, while only one QTL each was detected on the remaining five LGs 2, 4, 5, 8, and 9. Three QTL, *Qbsr-2.3*, *Qbsr-13.1*, and *Qbsr-14.4* were environment specific QTL detected only in one field environment. The remaining 15 BSR resistance QTL were detected in two or more field and/or greenhouse evaluation tests. A more detail description of these QTL is presented in Supplementary Table 3.

**Table 5:** Summary of Sclerotinia basal stalk rot resistance QTL identified in the HA 89/*Helianthus praecox* AB-QTL population.

QTL	Environment	Chromosome	Position (cM)	LOD range	Flanking markers		*R*^2^ range	Resistance allele source
					Left	Right		
*Qbsr-1.1*	Field, GH	1	27.5	4.16–5.47	C1_138804253	C1_138804178	8.76–10.83	*Helianthus praecox*
*Qbsr-1.2*	GH	1	35.2	5.22–5.35	C1_61152074	C1_77520719	9.15–10.01	*Helianthus praecox*
*Qbsr-1.3*	Field, GH	1	46.3–47.3	3.92–10.56	C1_36200423	C1_12280339	6.01–15.89	*Helianthus praecox*
*Qbsr-1.4*	Field	1	56.1	7.30–7.57	C1_128406849	C1_76087015	4.89–12.05	*Helianthus praecox*
*Qbsr-2.3*	Field	2	42.4	4.03	C2_57515151	C2_158778573	12.13	*Helianthus praecox*
*Qbsr-4.2*	Field	4	56.6	7.21	C4_82106550	C4_80109129	12.61	*Helianthus praecox*
*Qbsr-5.2*	GH	5	41.4	4.90–4.37	C5_135128592	C5_171556839	8.44–9.93	HA 89
*Qbsr-8.3*	Field	8	33.6	4.45	C8_76626672	C8_40519452	2.23–11.10	*Helianthus praecox*
*Qbsr-9.4*	GH	9	79.1	8.20	C9_184989573	C9_184989613	8.42	*Helianthus praecox*
*Qbsr-12.1*	Field	12	6.3–9.0	4.25–5.27	C12_20888727	C12_19391236	5.73–14.02	*Helianthus praecox*
*Qbsr-12.2*	GH	12	18.0–19.0	3.02–3.25	C12_64602921	C12_67215213	3.17–5.33	HA 89
*Qbsr-12.3*	Field	12	20.4–22.5	3.92–8.12	C12_66428651	C12_66487082	8.59–11.29	*Helianthus praecox*
*Qbsr-13.1*	Field	13	37.0	11.54	C13_128129098	C13_135991207	16.99	*Helianthus praecox*
*Qbsr-13.2*	GH	13	61.0	3.52–7.45	C13_171683116	C13_164101797	6.39–8.04	*Helianthus praecox*
*Qbsr-13.3*	GH	13	91.1	3.52–6.05	C13_95122315	C13_60411231	9.09–10.44	*Helianthus praecox*
*Qbsr-14.2*	Field	14	47.6–49.0	5.97–7.20	C14_151961331	C14_151961362	13.35–14.47	*Helianthus praecox*
*Qbsr-14.3*	GH	14	64.3	3.44–3.81	C14_48769986	C14_80841397	5.82–6.77	*Helianthus praecox*
*Qbsr-14.4*	Field	14	84.1	7.79	C14_130512048	C14_29518699	10.60	*Helianthus praecox*
*Qbsr-14.5*	Field, GH	14	99.5–100.0	3.14–3.33	C14_174055942	C14_141645099	2.21–10.10	HA 89

*LOD, log of odds; *R*^2^, phenotypic variation explained; and GH, greenhouse*.

## Discussion

Like most host species, genetic control of Sclerotinia BSR resistance in sunflower is quantitative and governed by additive gene effects. No complete resistance source is known so far in the cultivated or the crop wild relatives. Traditionally, most sunflower breeding programs evaluate germplasm lines under inoculated/naturally infected field conditions for BSR resistance sources from the primary gene-pool and select resistant lines in multi-parent crossing schemes for inbred line development. However, due to the narrow genetic base of the cultivated sunflower (Cheres and Knapp, 1998), recombination of BSR resistance alleles is limited, leaving the crop vulnerable to Sclerotinia infection. Despite several known sources of Sclerotinia resistance in the wild sunflower gene-pools (for review, see Seiler et al. (2017b)), detailed analysis of resistance at the genomic level has not been attempted until recently (Qi et al., 2016, 2018; Talukder et al., 2019a,b, 2021). Recent advances in sunflower genomic resources (Badouin et al., 2017)^2^ coupled with decreasing costs of next-generation sequencing (NGS) technology has enabled the discovery and tagging of DNA markers linked to the quantitative genetic variations for Sclerotinia resistance in sunflower. Introgression of Sclerotinia BSR resistance from wild *H. praecox* species into a cultivated sunflower background has been accomplished (Talukder et al., 2019a) and sunflower germplasm lines possessing higher level of BSR resistance have been developed and publicly released for use in BSR resistance breeding programs (Talukder et al., 2019b). Our current project is a follow-up research effort to molecularly dissect the transferred BSR resistance with respect to distribution, magnitude, and nature of expression under different growing environments.

The phenotypic responses to the BSR resistance in the AB-QTL population showed continuous distribution in both field and greenhouse evaluations (Figure 1; Supplemental Figure S1), typical nature for a quantitative trait controlled by numerous genes (Young, 1996). Despite seasonal variation in host resistance response to BSR disease, Spearman’s rank correlation between DI scores of the two seasons was highly significant (Figure 1D), validating the repeatability of the field screening trials. Similar seasonal variations were also observed when BSR field screening trials were conducted at the Carrington field site for a different sunflower mapping population (Talukder et al., 2021). Analysis of variance revealed that both genotype and genotype × environment interactions are highly significant for BSR DI measured in the field trials (Table 1). However, the variance component for the genotypes was much higher than the variance component for the genotype × environment interactions, signifying the greater contribution of the genetic makeups of the AB-QTL progeny lines to BSR resistance. This assumption was further supported by the moderately high (0.66) broad-sense heritability (*H*^2^) that measures the contribution of genetic factors in the observed phenotype.

The evaluation of the AB-QTL population in the greenhouse is robust and uniform since sunflower plants are inoculated individually and a micro-climate conducive to the BSR disease development is maintained where disease avoidance is not possible (Underwood et al., 2021). Two traits, AUDPC and DR were measured in the greenhouse trials to assess BSR resistance in the AB-QTL progeny lines. As expected, the phenotypic distribution of both the traits were also continuous in the AB-QTL population (Figures 1E, F). However, unlike BSR DI measured in the field, the AUDPC and DR data were skewed toward higher AUDPC and lower DR values in the greenhouse. The apparent inconsistency might be due to the high disease pressure put on the young sunflower seedlings in the greenhouse trials. Interestingly, the Spearman’s rank correlation among the greenhouse measured traits and DI measured in the field were significant at varying levels (Table 2), suggesting a collinearity between the field and greenhouse evaluation of the HA 89/*H. praecox* AB-QTL population to some extent. Strong correlation between greenhouse and field evaluations have been previously observed when a panel of cultivated sunflower lines were evaluated for BSR disease (Underwood et al., 2021). Broad-sense heritability of the AUDPC (0.73) and DR (0.70) measured in the greenhouse test were also comparable to the heritability (*H*^2^ = 0.66) of BSR DI measured in the field. It is also important to note that disease evaluations in this study were performed using a single isolate of *S. sclerotiorum*. Further research will be required to determine if genotype-isolate interactions are relevant to sunflower BSR resistance and to ensure that resistant genotypes and QTL identified in this study provide broad-spectrum resistance to a range of *S. sclerotiorum* isolates.

The linkage map developed for the HA 89/*H. praecox* AB-QTL population contained a total of 1,710 loci mapped on 17 sunflower chromosomes, which is not a very dense linkage map as one would expect from a biparental sunflower mapping population derived from two highly diverse parental lines (wild vs. cultivated). Segregation distortion is a common feature in many interspecific crosses (Zamir and Tadmor, 1986). In a backcross population involving wild species, most often the distorted ratios favor alleles from the cultivated recurrent parent (Rieseberg and Carney, 1998). Despite a high level of polymorphism between the parents at the DNA marker level, many of the markers were eventually removed from the linkage analysis due to severe segregation distortion. The total length of the current linkage map for the HA 89/*H. praecox* AB-QTL population was 1,802.95 cM, which is comparable to the SNP marker based high-density linkage maps reported in sunflower (Bowers et al., 2012; Talukder et al., 2014a, 2016, 2020, 2021; Gao et al., 2017; Zhou et al., 2018).

Due to the presence of significant genotype × environment interactions, QTL analyses of the Carrington field data were performed individually for each environment, as well as, in combination using phenotypic BLUPs (the best linear unbiased predictors) of two seasons DI data. The AUDPC and DR data collected in the greenhouse were also analyzed independently. A total of 19 QTL associated with BSR resistance on nine sunflower chromosomes were detected in the current study. Similarly, an earlier study also reported a large number of BSR resistance QTL identified in a population derived from a wild sunflower species (Talukder et al., 2021). The QTL alleles conferring BSR resistance in the AB-QTL population were mostly derived from the wild *H. praecox* parent (~84%), which is consistent with the previous observations where *H. praecox* derived introgression lines showed higher levels of resistance to Sclerotinia BSR disease than the cultivated sunflower lines (Talukder et al., 2019b). Among the 19 detected QTL, nine of them were detected in the field evaluation tests alone, including the QTL on LG 13, *Qbsr-13.1*, which explained the largest proportion of the phenotypic variance (16.99%) among all the QTL detected in this population. Similarly, seven QTL were detected only in the greenhouse test on LGs 1, 5, 9, 12, 13, and 14, of which the BSR resistance alleles for *Qbsr-5.2* and *Qbsr-12.2* were derived from the cultivated parent. Despite strong Spearman’s rank correlation between field and greenhouse traits, only three BSR resistance QTL were detected in both field and greenhouse environments on LGs 1 and 14 of the sunflower genome. Among these three ubiquitous QTL, *Qbsr-1.1* and *Qbsr-1.3* were detected on LG1 with BSR resistance contributing alleles derived from the wild *H. praecox* source. Both these QTL were mapped with high LOD values and explained moderate phenotypic effect for the BSR resistance in the AB-QTL population (Supplementary Table 3). Due to their stability across environments and novel resistance allele source, these two QTL would be the first choice to use in MAS breeding program designed for introgression of BSR-resistant into elite sunflower lines.

A review of published works revealed that Sclerotinia BSR resistance QTL were previously mapped on 15 of the 17 LGs in the sunflower genome except on LGs 12 and 13 (Davar et al., 2010; Talukder et al., 2016, 2021; Paknia et al., 2020). However, in our current study, BSR resistance QTL were mapped to two additional LGs 12 and 13 that were not previously reported in any published works (Supplementary Table 4). The QTL, *Qbsr-12.1*, *Qbsr-12.3*, *Qbsr-13.1*, *Qbsr-13.2* and, *Qbsr-13.3* mapped on these LGs had their BSR resistance alleles derived from the *H. praecox* and can be considered as a novel QTL source. Sequence comparison of the flanking markers in the sunflower genome revealed that *Qbsr-4.2* was mapped in the same region, where the BSR resistance QTL *Qbsr-4.1* was detected in the HA 441/RHA 439 population (Talukder et al., 2016). Similarly, the *Qbsr-8.3* and *Qbsr-14.3* QTL mapped in the current study on LGs 8 and 14, respectively, were located on the same genomic regions as *Qbsr-8.2* and *Qbsr-14.1* in the HA 89/*H. argophyllus* AB-QTL population (Talukder et al., 2021). The sunflower candidate gene, *HaCOI1*-2 paralogous to the *A. thaliana COI1* gene (Talukder et al., 2014c) is present in the genomic region flanking by the significant SNP markers associated with BSR resistance QTL, *Qbsr-14.3* on LG14. The other sunflower candidate gene, *HaCOI1*-1 significantly associated with Sclerotinia BSR (Talukder et al., 2014c) and HR (Filippi et al., 2020) resistance is located in the genomic region flanking by the SNP markers associated with the *Qbsr-14.5* QTL mapped in the current study. Except for *Qbsr-4.2*, *Qbsr-8.3*, *Qbsr-14.3*, and *Qbsr-14.5*, all other QTL mapped in the current study are so far uniquely mapped to the HA 89/*H. praecox* AB-QTL population with BSR resistance alleles derived from the *H. praecox* parent and considered as a novel QTL source. A comprehensive comparison of BSR resistance QTL mapped in the RIL population derived from the cross PAC2/RHA266 (Davar et al., 2010; Amouzadeh et al., 2013; Paknia et al., 2020) with QTL detected in the current study could not be performed due to lack of sequence information of the flanking markers used in those studies.

Introgression of BSR resistance from wild species into cultivated sunflower background can certainly broaden the genetic diversity in the primary gene-pool of the crop (Qi et al., 2016, 2018; Talukder et al., 2019b,c). The use of the AB-QTL population approach has made a significant contribution in unraveling the valuable alleles from wild species sources (Wang and Chee, 2010; Talukder et al., 2021). It is clear that Sclerotinia BSR resistance in sunflower is a complex trait and controlled by many small-effect genes. QTL mapping provides a framework for marker-assisted selection of such a complex disease resistance trait. Molecular markers linked to the significant BSR resistance QTL identified in this study will provide a valuable resource in marker-based recurrent selection to develop BSR resistant sunflower (Johnson, 2001; Bernardo, 2004).

## Data Availability Statement

The datasets presented in this study can be found in online repositories. The names of the repository/repositories and accession number(s) can be found at: European Variation Archive (EVA) under the Project: PRJEB50764 and Analyses: ERZ5072953.

## Author Contributions

LQ and ZT conceived and designed the experiments. LQ developed the population and contributed to writing—review and editing. ZT, WU, CM, GS, LQ, XC, and XL performed the experiments. ZT, WU, and LQ analyzed the data. ZT prepared the original draft. All authors contributed to the article and approved the submitted version.

## Funding

This research was supported by the USDA–ARS National Sclerotinia Initiative, grant number 3060-21220-031-00D and the USDA–ARS CRIS project no. 3060-2100-043-00D.

## Conflict of Interest

The authors declare that the research was conducted in the absence of any commercial or financial relationships that could be construed as a potential conflict of interest. Mention of trade names or commercial products in this report is solely for providing specific information and does not imply recommendations or endorsement by the USDA. The USDA is an equal opportunity provider and employer.

## Publisher’s Note

All claims expressed in this article are solely those of the authors and do not necessarily represent those of their affiliated organizations, or those of the publisher, the editors and the reviewers. Any product that may be evaluated in this article, or claim that may be made by its manufacturer, is not guaranteed or endorsed by the publisher.

## Footnotes

^1^ https://www.heliagene.org/HanXRQ-SUNRISE

^2^ https://www.sunflowergenome.org

## Supplementary Material

Supplementary Figure 1 Heat map showing the performance of 174 individuals of the HA 89/*H. praecox* advanced backcross population evaluated for Sclerotinia basal stalk rot resistance in the greenhouse and field environments. Area under disease progress curve (AUDPC) and disease rating (DR) were measured in the greenhouse in 2018, and disease incidence (DI) were measured in the Carrington, ND field in 2017 and 2018.
