# Supplementary figures and images for: Genomic Insights Into Sclerotinia Basal Stalk Rot Resistance Introgressed From Wild Helianthus praecox Into Cultivated Sunflower (Helianthus annuus L.)
Source: Front Plant Sci. 2022 May 18;13:840954. doi: 10.3389/fpls.2022.840954 (PMC9158519; doi:10.3389/fpls.2022.840954)

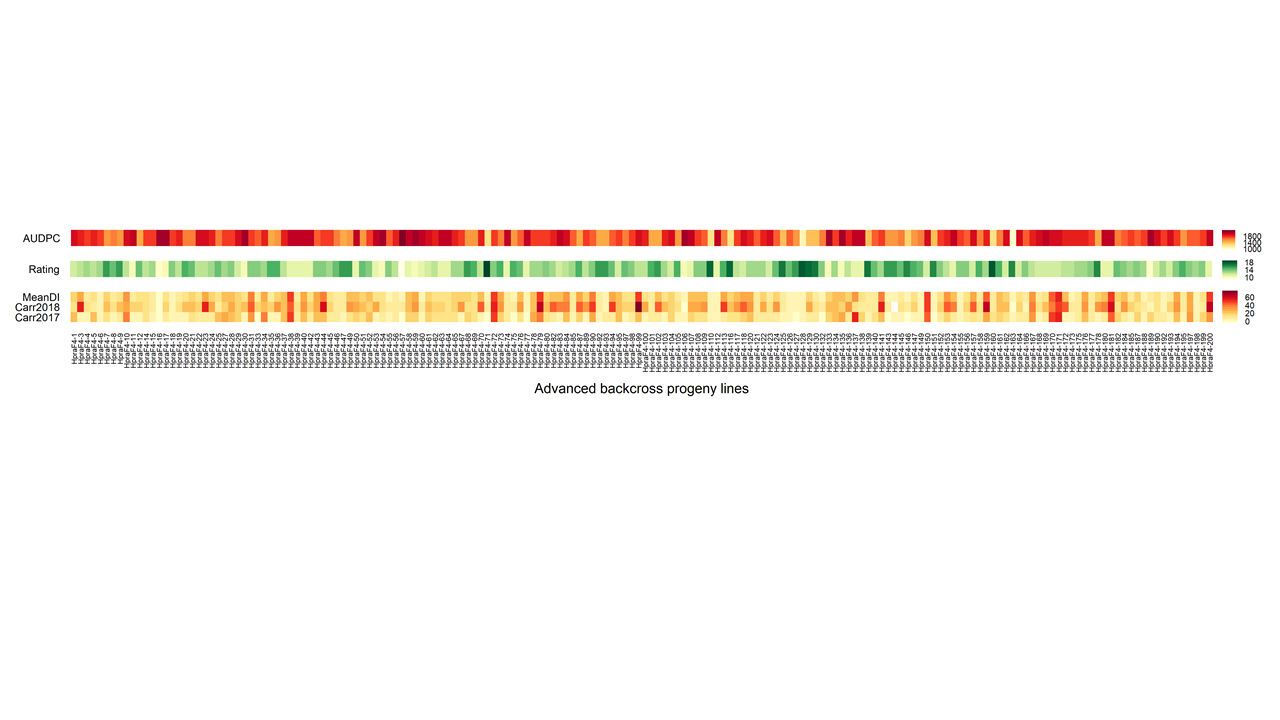

Supplement: Supplementary Figure 1 — Heat map showing the performance of 174 individuals of the HA 89/H. praecox advanced backcross population evaluated for Sclerotinia basal stalk rot resistance in the greenhouse and field environments. Area under disease progress curve (AUDPC) and disease rating (DR) were measured in the greenhouse in 2018, and disease incidence (DI) were measured in the Carrington, ND field in 2017 and 2018. [file Image_1.TIF]
